# Systemic moxifloxacin vs amoxicillin/metronidazole adjunct to non-surgical treatment in generalized aggressive periodontitis

**DOI:** 10.4317/medoral.20552

**Published:** 2015-06-02

**Authors:** Esra Guzeldemir-Akcakanat, Cem-Abdulkadir Gurgan

**Affiliations:** 1 Department of Periodontology, Faculty of Dentistry, Kocaeli University, Kocaeli, Turkey; 2 Department of Periodontology, Faculty of Dentistry, Erciyes University, Kayseri, Turkey

## Abstract

**Background:**

The objective of this randomized clinical study was to evaluate the effect of systemic administration of moxifloxacin compared to amoxicillin and metronidazole, combined with non-surgical treatment in patients with generalized aggressive periodontitis (GAgP) in a 6-month follow-up.

**Material and Methods:**

A total of 39 systemically healthy patients with GAgP were evaluated in this randomized clinical trial. Periodontal parameters were recorded at the baseline during the 1st, 3rd and 6th month. Patients received either 400 mg of moxifloxacin per os once daily or 500 mg of metronidazole and 500 mg amoxicillin per os three times daily for 7 days consecutively.

**Results:**

No significant differences between groups were found in any parameters at the baseline. Both groups led to a statistically significant decrease in all clinical periodontal parameters compared to the baseline (PI, *p*<0.001 and GI, PD, BOP, CAL, *p*<0.01). There were no differences between the 1st and 3rd months or the 3rd and 6th months for clinical parameters in the groups. Also, no intergroup difference was observed in any parameters at any time, except the gingival index at 6th months.

**Conclusions:**

Systemic administration of moxifloxacin as an adjunct to non-surgical treatment significantly improves clinical outcomes and provides comparable clinical improvement with less adverse events to that of combination of amoxicillin and metronidazole in the treatment of GAgP.

**Key words:** Aggressive periodontitis, amoxicillin, metronidazole, moxifloxacin, nonsurgical periodontal debridement.

## Introduction

Aggressive periodontitis (AgP) is an inflammatory periodontal disease, which is complex, multi factorial, and destructive.

AgP has been treated like other forms of periodontitis. The established treatment of periodontitis involves cause-related therapy, which includes: maintenance of oral hygiene, scaling and root planing (SRP), and surge-ries of affected sites. Contrary to gingivitis and chronic periodontitis, mechanical non-surgical treatment does not always provide expected results in the treatment of AgP. In these cases, antibiotics may be used as an adjunct to treatment for eliminating or reducing the number of specific microorganisms and improving clinical parameters when compared with SRP alone (1-4). The American Academy of Period ontology (AAP) and the European Federation of Period ontology (EFP) were reported that patients with GAgP may have benefit from the adjunctive systemic administration of antibiotics (1,5,6).

Amoxicillin is a moderate spectrum; bacteriolytic β-lactam antibiotic, and metronidazole is active against anaerobic bacteria. SRP combination with metronidazole and amoxicillin therapy was found to be more effective in suppressing *P.g*. and eradication of *A.a*. and preventing re-colonization of *A.a*. because of the synergistic effect of this combination and their wide spectrum of activity. It was found to be superior to azithromycin, doxycycline, and metronidazole in the treatment of GAgP.

Moxiflocaxin is a fourth generation fluoroquinolone antibiotic and exhibits good tissue penetration and high oral bioavailability. It has improved activity against Gr (-), aerobic and anaerobes and has good activity against putative periodontal pathogens located within biofilm or intracellulary (7).

To the best of our knowledge, there is no study that evaluates the effect of moxifloxacin adjunct to SRP in the treatment of GAgP.

The aim of this study was to evaluate the impact of adjunctive systemic moxifloxacin; compared to the use of adjunctive systemic amoxicillin and metronidazole during full-mouth SRP based on the success of the treatment of patients with GAgP along with a 6-month follow-up.

## Material and Methods

This study was a single-center, randomized, parallel-design clinical trial with 6 month follow-up. The study protocol was approved by the Ethics Committee of the Medical Faculty of Kocaeli University (KOU KAEK 5/9). This clinical trial is registered; the identifier number is NCT02223702 (www.clinicaltrials.gov). The study protocol explained to the patients. From patients who willing to participate to the study, written informed consent was obtained and included the study.

The periodontal diagnosis of subjects with GAgP was performed according to the 1999 International World Workshop for a Classification of Periodontal Diseases and Conditions. Patients were included if they were between 18 and 35 years of age and otherwise healthy. Subjects were excluded if they had any known systemic diseases or conditions that can/could influence the periodontal status, allergies to quinolones, penicillin or metronidazole, a history of antibiotic therapy, or periodontal treatment within the preceding six months.

- Periodontal Examination

The full-mouth clinical periodontal measurements were recorded at 6 sites per tooth, including plaque index (PI), gingival index (GI), probing depth (PD), bleeding on probing (BOP) and clinical attachment loss (CAL).

- Non surgical treatment

Prior to treatment, all subjects had gone through motivation sessions for oral hygiene. Following periodontal measurements, full-mouth supra gingival scaling using an ultrasonic scaler and polishing was performed. A toothbrush, toothpaste and an inter proximal toothbrush were provided to all subjects. One week later, the patients were examined for plaque accumulation and oral hygiene. The patients who cannot maintain proper oral hygiene were excluded from the study. Only 39 patients fulfilled the qualification criteria for enrollment for the present study.

Subjects were randomly assigned to receive one of the two treatment groups. The moxifloxacin (MXF) group received SRP and adjunctive systemic antibiotic, 400 mg MXF (1x1, 7 days). The amoxicillin and metronidazole (AMX+MET) group received a combination of 500 mg of amoxicillin and 500 mg metronidazole (3x1, 7 days). The subjects were instructed to take the first dose of the antibiotics in the morning of the first session. SRP were performed during 2 consecutive days in 24-h under the local anesthesia. On each day, SRP were performed in 2 quadrants using ultrasonic scalers and manual instruments. The endpoint of SRP was a tactile, smooth root surface.

Patients used a 0.2% chlorhexidine digluconate rinse (2x1, 30 days) and brushed their teeth by toothbrushes and inter proximal toothbrushes twice a day. Patients asked to report any adverse events and side effects of the anti microbial agents.

Subjects were monitored one week after the second SRP session. At this session, antibiotic intake and adverse events were questioned; oral hygiene was controlled. Subjects were screened at 1, 3 and 6 months after completion of SRP. During these sessions, periodontal indices and any medical history change; especially whether antibiotic therapy had been prescribed for any reason was recorded.

- Statistical Analysis

The reliability of continuous variables was expressed as the SD of the differences divided by 2. According to the reliability analysis for PD and CAL, the measurement errors were 0.13 and 0.11, respectively. Cohen’s K was employed to describe the reliability of discrete PI and GI values. Based on the duplicate measurements, the kappa values of PI and GI were 0.76±0.04 and 0.86±0.05, respectively.

Depending on the normality in the distribution of the clinical parameters, Mann-Whitney-U and independent T-test were used for the differences between the groups. The changes in clinical parameters among different evaluation periods for each group were analyzed using Friedman and the repeated measure ANOVA test, where applicable. Additionally, when the p value from the Friedman test was statistically significant, multiple comparison tests were used to ascertain which evaluation time point differed from the others.

## Results

**Figure 1:**
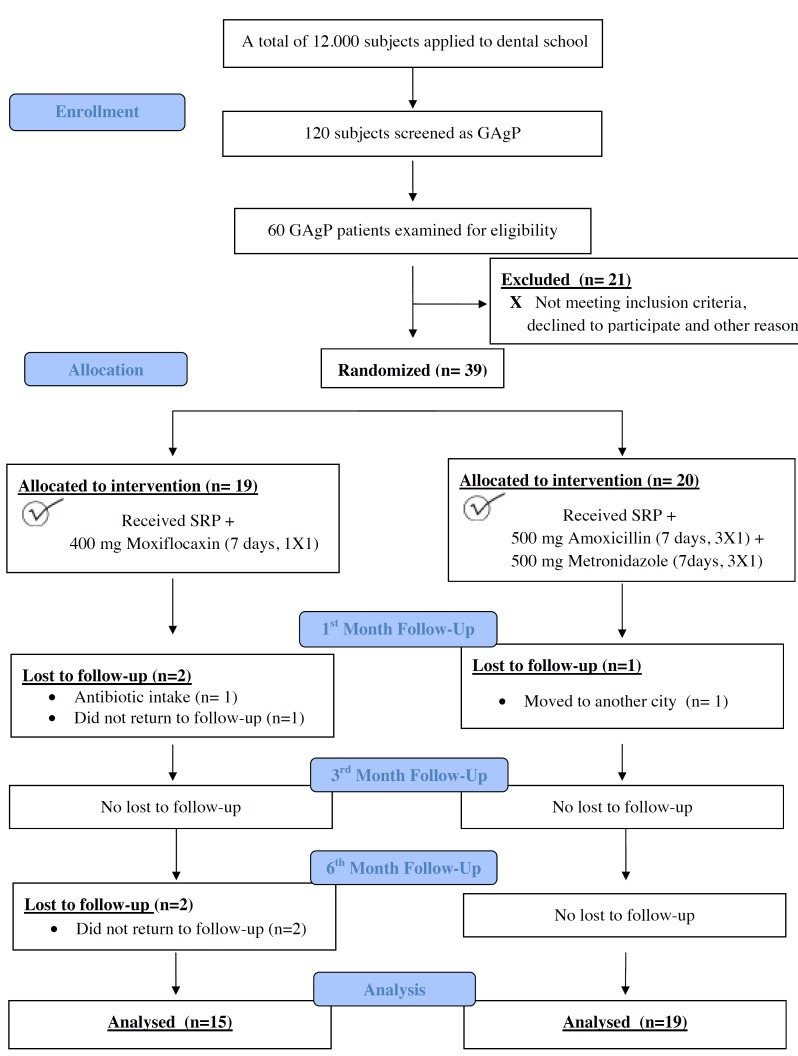
Flow diagram of the study according to CONSORT 2010.

Figure 1 shows flow diagram of the present study. A total of 39 subjects were enrolled into the present study; baseline demographic variables and clinical periodontal parameters are shown in  Table 1.

**Table 1:**
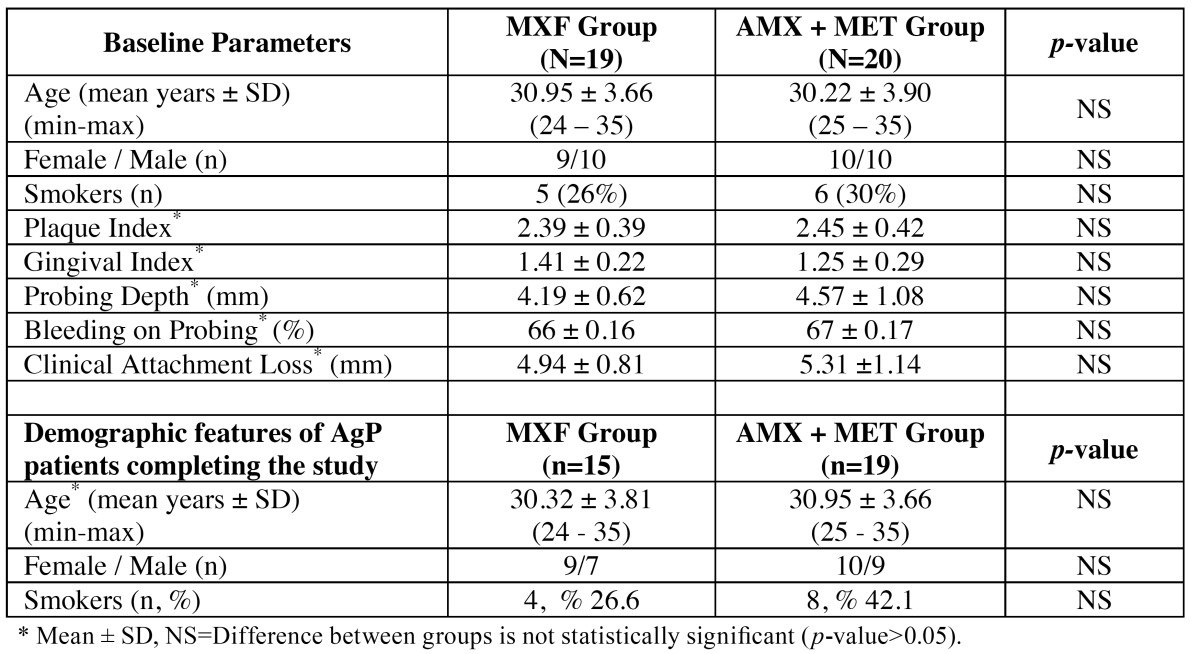
Demographic and clinical characteristics of the GAgP patients.

While one subject from AMX+MET group was withdrawn at 1st month visit, four subjects from MXF group were withdrawn at 1st and 6th month visits. Fifteen subjects in the MXF group (7 males and 9 females; mean age, 30.32±3.81 years; range, 24-35 yrs) and 19 subjects in the AMX+MET group (9 males and 10 females; mean age, 30.95±3.66 yrs; range, 25-35 years) completed the study ( Table 1). There were no significant differences between the groups at any point related to age, gender, and smoking status. (*p*>0.05) ( Table 1).

Each subject of the MXF group received 7 tablets of MXF 400 mg and each subject of the AMX+MET group received a total of 21 tablets of AMX 500 mg and MET 500 mg.

While none of the test subjects reported any adverse event associated with MXF, two subjects reported a stomachache and one subject reported gastrointestinal problems related to AMX+MET intake.

Both groups demonstrated statistically a significant decrease in all clinical periodontal parameters at the end of the 1st, 3rd and 6th months compared to the baseline (PI; *p*<0.001 and GI, PD, BOP, CAL, *p*<0.01,  Table 2 and  Table 3).

**Table 2:**
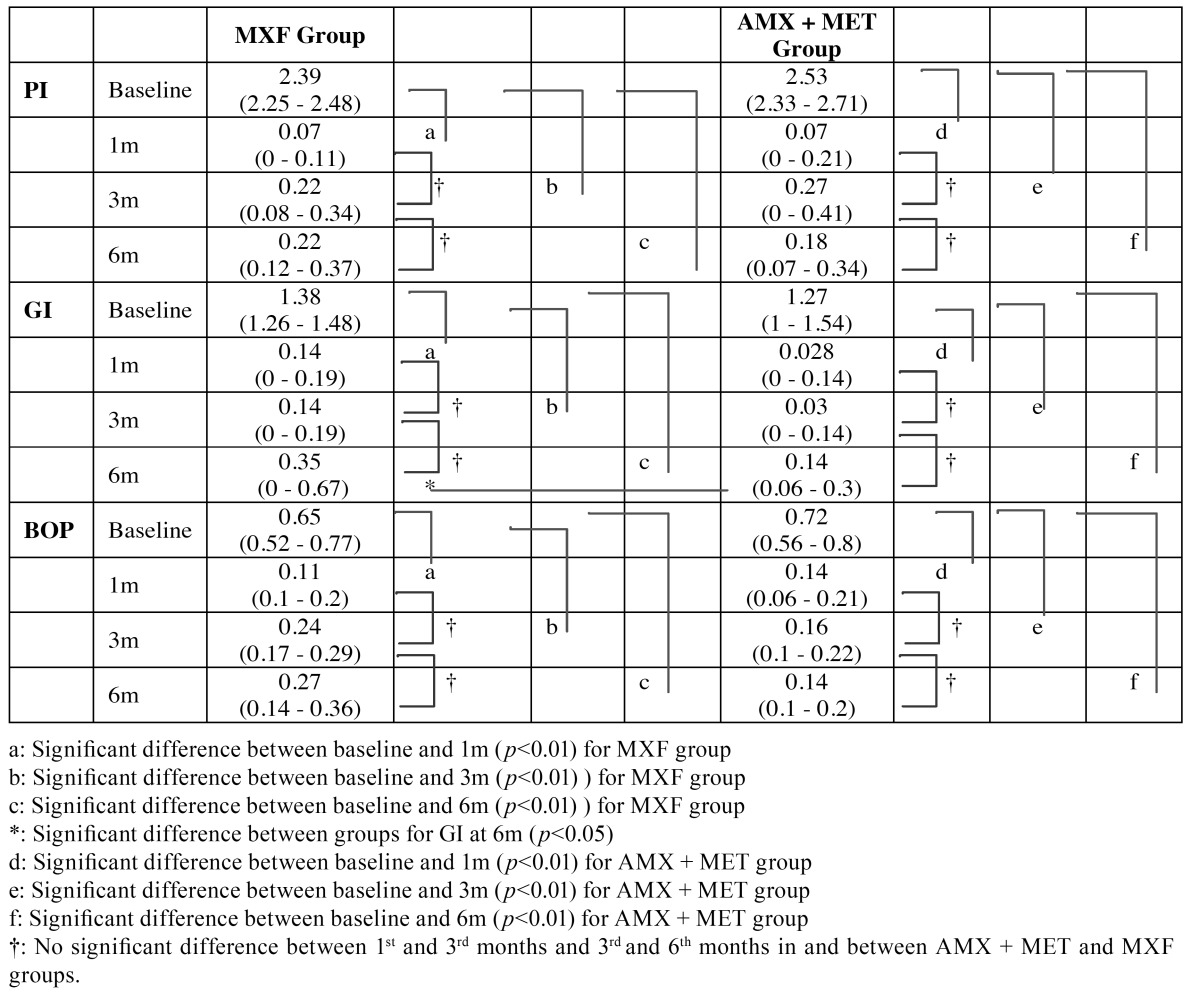
Median (25th-75th percentiles) values for plaque index (PI), gingival index (GI) and bleeding on probing (BOP) of MXF and AMX+MET groups for each recalls.

**Table 3:**
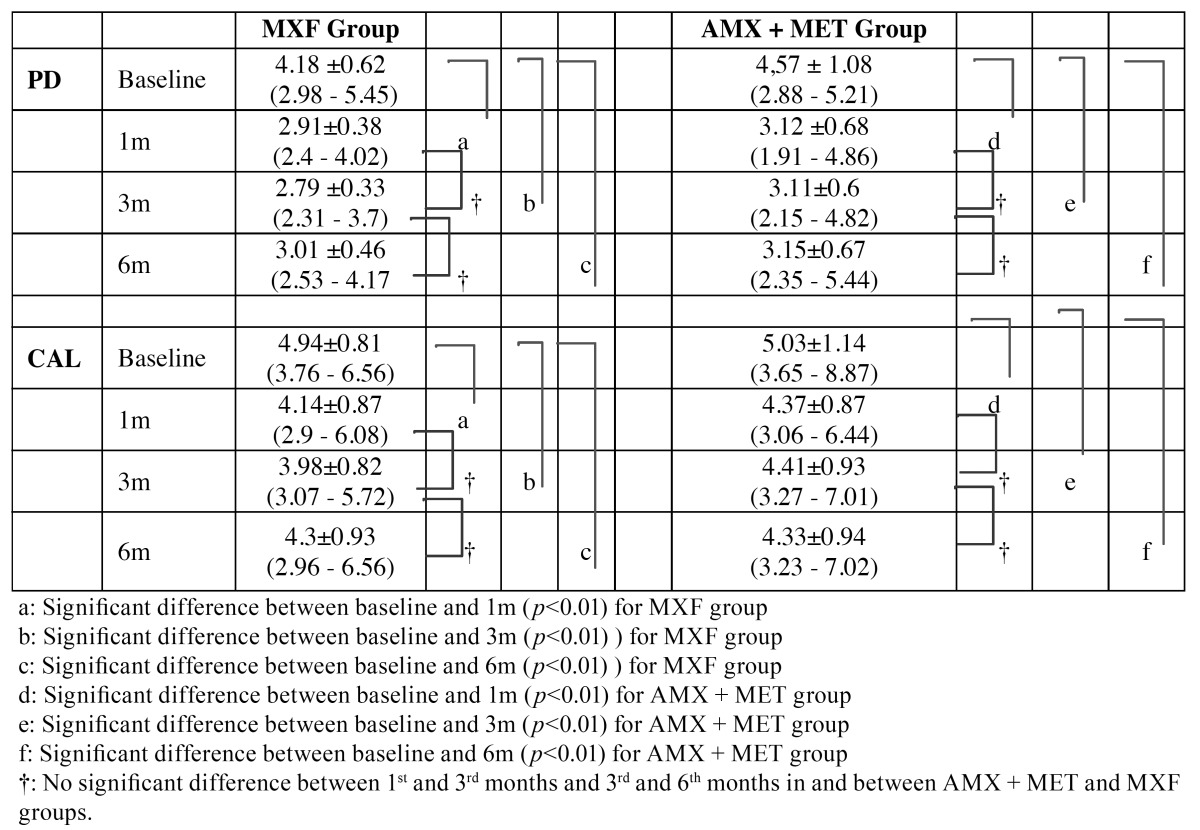
Mean ± standard deviation (minimum-maximum) values for probing depth (PD) and clinical attachment loss (CAL) of each recall.

There were no significant differences between the 1st and 3rd, and 3rd and 6th month in the groups whereas none of the periodontal parameters showed intergroup differences in any time points (*p*>0.01), except GI at 6th months (*p*<0.05) ( Table 2 and  Table 3)

As shown in table 3, both groups demonstrated statistically a significant decrease in PD and CAL at the 1st, 3rd and 6th month compared to the baseline (*p*<0.01). While the greatest reduction in the mean PD was seen at the 3rd month, compared to the baseline in both groups (*p*<0.01), but this difference was not statistically significant compared to the 1st and the 6th month. The greatest reduction in the mean CAL was seen during the 1st month in the MXF group and in the 3rd month in the AMX+MET group compared to the baseline (*p*<0.01). However, these differences were not significant compared to other follow-up visits. No difference was found between groups at any point in 6 months.

The mean PD reduction and the mean clinical attachment gain at the 6th month were 1.17±0.16 mm and 0.64±0.12 mm, respectively, in MXF group and 1.42±0.41 and 0.70±0.20 mm, respectively, in AMX+MET group. No significant differences were observed between groups.

The changes as percentage in PD and CAL are also evaluated under 3 categories, as <4 mm (shallow), 4-6 mm (moderate) and >6 mm (severe) (8). Percentage of PD was increased to 27.56% in pockets initially <4 mm in MXF group and 33.61% in AMX+MET group ( Table 4), and no difference was seen between groups. Percentage of PD was decreased by 14.99% in pockets initially 4-6 mm, 12.65 in pockets initially >6 mm in MXF group, and 14.75% and 17.80% % in AMX+MET group, respectively ( Table 4) and no differences were seen between groups.

**Table 4:**
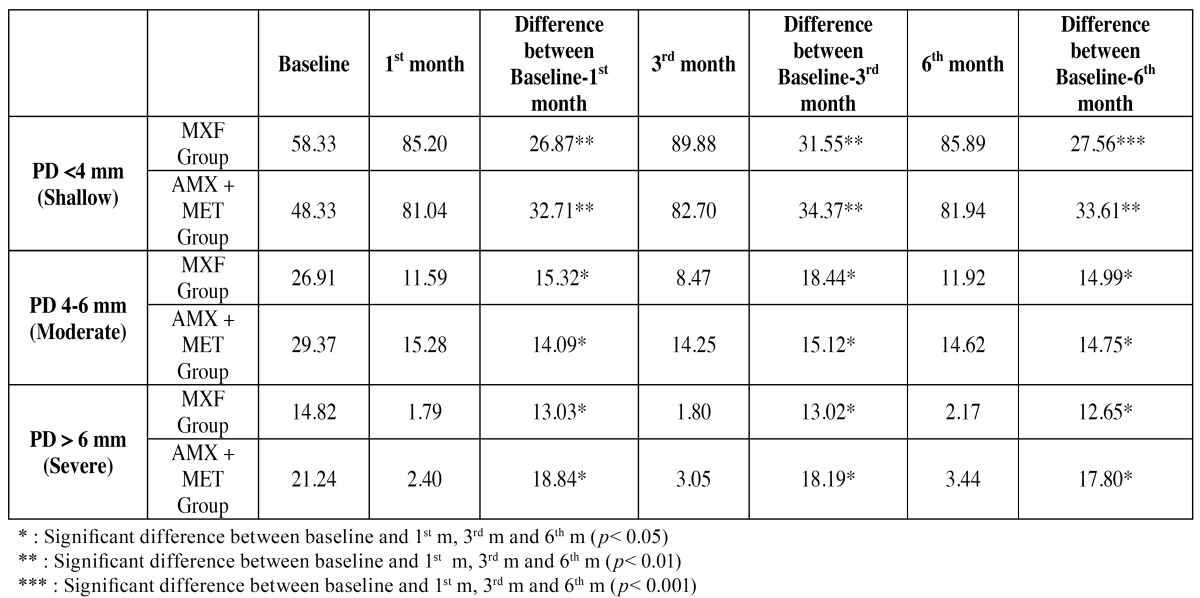
Alterations of probing depth (PD) (a) and clinical attachment loss (CAL) (b) categories as percentage among recall periods for the groups. (a) Probing depth (PD) categories (%).

Percentage of CAL was increased by 46.70% in pockets initially <4 mm in MXF group and by 42.10% in AMX+MET group ( Table 5), and there was no significant difference between the groups. Percentage of CAL was decreased by 26.70% in CAL initially >6 mm, 20.00% in CAL initially 4-6 mm in MXF group, and 26.30% and 15.8% in AMX+MET group, respectively. No differences were observed between the groups; however, there was a significant difference between the 1st and the 3rd month in CAL 4-6 mm category in MXF group (*p*<0.05).

**Table 5:**
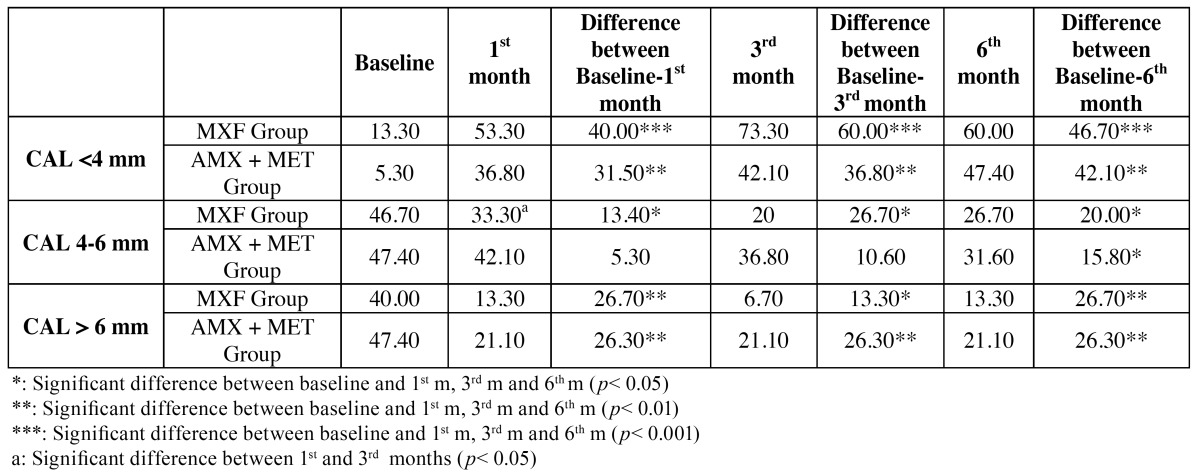
Alterations of probing depth (PD) (a) and clinical attachment loss (CAL) (b) categories as percentage among recall periods for the groups. (b) Clinical attachment loss (CAL) categories (%).

There were no differences between groups in any categories at any time.

## Discussion

To our knowledge, this study was the first clinical study, which evaluates the effects of systemic moxifloxacin compared to the adjunctive systemic amoxicillin and metronidazole as an adjunct to nonsurgical treatment in the treatment of patients with GAgP. On the basis of the present findings, it can be concluded that adjunctive moxifloxacin provides comparable clinical improvement to that of the combination of amoxicillin and metronidazole in the treatment of GAgP.

All periodontal clinical parameters were dramatically decreased in both groups at the 1st month compared to the baseline and all patients maintained good hygiene and post-treatment follow-ups. In the present study, although no effort was made to control the smoking; smokers were equally distributed between groups and the results did not change (data not shown).

Management of AgP is always challenging for clinicians since every case is unique and there are no established treatment guidelines or protocols. In the treatment of AgP, a number of anti microbial regimens have been investigated, which aim to potentiating the effects of SRP. It was shown that non surgical treatment together with systemic use of antibiotics in AgP yields better clinical results and less surgery needs (9-11) when comparing non surgical treatment alone (1,2,12).

In the present study, there were no differences in, and between the groups at any time. Then, we categorized the parameters as slight, moderate, and severe ( Table 4 and  Table 5). There was also no difference between the groups. However, when each patient was evaluated as a separate entity, it was found that the alterations in the frequencies of CAL categories were significantly different at all times in both groups.

The mean for the full-mouth PD reduction and the clinical attachment gain were better (13) or comparable (4,8,10-12) to those reported in patients with GAgP at the 6th month. However, it is not convenient to make direct comparisons between the studies due to discrepancies among the study methodologies.

In the treatment of AgP, different antibiotic regimens, length of antibiotic therapy, timing of the administration, different dosages, evaluation parameters and clinical outcome assessments have been studied, however there is no consensus or definite conclusion, yet (2,9). As stated by Mombelli *et al*. (9); “Useful antibiotic regimes for distinct clinical or microbiological conditions could not be clearly identified based on available evidence,” “The optimal timing for antibiotic administration is still controversial”, hence, “There are no evidence based guidelines for the use of systemically administered antibiotics (14).” 

Different amoxicillin and metronidazole dosages used by the researchers in the studies. In the present study, 500 mg of both amoxicillin and metronidazole, three times a day were prescribed for each person to reach a minimum effective concentration of antibiotics in gingival crevicular fluid and blood.

The timing of the antibiotic regime is still unclear, however, based on studies and reviews, Consensus Report of the Sixth European Workshop on Period ontology concluded that antibiotic intake should start on the day of non-surgical treatment (15).

Duration of the antibiotic regime also changes between the studies. Nevertheless, to date, there is no clear statement about the duration of the antibiotic use. It is concluded that the SRP should be carried out in the shortest time possible, preferably less than 7 days in AgP (15,16). Hence, in the present study, antibiotic administration was started after the initial periodontal treatment; in the morning of the same day of SRP, and the treatment was completed in 24 hours, using 7-day systemic antibiotic regimes for both groups.

Moxifloxacin is a fourth-generation fluoroquinolone with a broad spectrum of activity against microorganisms and pathogens with resistance to penicillin, ma-crolides, and tetracyclines. It contains a C-8 methoxy substitute that increases bactericidal activity and there is a marked the time-kill kinetics and post-antibiotic effect (17). Moxifloxacin is effective and generally very well tolerated by patients and the reported side effects are very low. It is expected that the patient’s compliance might be enhanced since prescribing a single-dose per day is possible, due to its pharmacokinetics.

Efficacy of moxifloxacin in dental research is mostly evaluated in the treatment of odontogenic abscesses or infiltrates and promising in vitro activity against odontogenic pathogens was revealed over amoxicillin-clavulanic acid, clindamycine, doxycycline and penicillin. Moxifloxacin has a favorable bacterial activity against putative periodontal pathogens including *P.g*., *A.a*. and *T. forsythia* (7,18). In an in vitro study, the activity of moxifloxacin was compared with ofloxacin and doxycycline against single-species biofilms of two *P.g*. and two *A.a*. strains and a multi species biofilm consisting of 12 species and a topical formulation of moxifloxacin as an adjunct to mechanical treatment is suggested (19). The good penetration of moxifloxacin into soft and hard tissues in Wistar rats was also shown (20). Moxifloxacin was found to be comparable to that of amoxicillin-clavulanate however superior to those of clindamisin, metronidazole, doxycycline or penicillin (21-23).

In a study by Müller *et al*. (7), it showed that A.a. strains were highly susceptible to fluoroquinolones, ciprofloxacin and moxifloxacin. Some of the adjunctive antibiotics are secreted in saliva in insufficient concentrations to inhibit *A.a*. Moxifloxacin seems to be secreted in saliva at higher levels than in plasma and may also concentrate at the site of infection since it penetrates polymorphonuclear granulocytes and epithelial cells (7).

In the present study, to potentiate the outcomes of non surgical periodontal treatment, SRP performed in 24-h period in combination with chlorhexidine digluconate mouth rinse and systemically administered antibio tics by reducing the number of periodontopathogen not only from periodontal pockets but also from their other habitats such as saliva, the tongue, and by retarding re-colonization of the bacteria. AgP patients might have benefits from this approach.

The present study is the use of only one type of antibiotic with less amounts of tablets; instead of two different types of antibiotics with a lot more tablets taken. This treatment alternative might cause fewer side effects with similar clinical outcomes and enhance patient compliance. In the contrary to previous reports, three patients who use AMX+MET, reported adverse effects (4,12). However, other studies are reported varying side effects related to AMX+MET (11,24,25). There is no report regarding adverse effects of moxifloxacin in periodontal treatment.

One would consider why a control group (without antibiotics) did not think to be included in the present study. This might be a one of the limitations of the present study. However, as we summarized and discussed in this section, the antibiotic used as an adjunct to SRP is approved with research and accepted with consensus reports released by EFP and AAP in the treatment of GAgP. Hence, we thought that it would not be ethical to monitor the non surgical treatment of patients with GAgP without an adjunct antibiotic. While the effect of adjunctive AMX+MET in the treatment of GAgP is well defined (4,11,12,26), antibiotic combination of AMX+MET was considered as control.

Another limitation of the present study would be the lack of micro biological evaluation. There are two major indications for micro biological testing in Period ontology; first, to detect sub gingival microbial flora for accurate diagnosis. It was stated that all patients with GAgP may not have an identical and specific microbial profile related with GAgP (27). Moreover, sub gingival microbial factors in GAgP do not seem different from patients with chronic periodontitis. Prevalence and behaviors of periodontopathogens may not adapt to diffe-rent populations and ethnicities (28,29). It was reported that *A.a*.-positive patients had no specific benefit from AMX+MET (24), which means combinations or specific antibiotic regimens may not work in all cases (14). On the other hand, Heller *et al*. (26) emphasized inter-individual varieties of periodontopathogens in the sub gingival microbial flora of patients with GAgP and concluded that not all GAgP patients had greater microbial benefits from AMX+MET combination. The patients had species associated with GAgP were more affected from this combination.

Second, was to determine the antibiotic susceptibility/resistance of periodontopathogenic profiles for proper treatment. It was noted that European and South American countries have significant differences in their antibiotic susceptibility profiles of periodontal bacteria (30). van Winkelhoff *et al*. (30) concluded that “it may not be possible to develop uniform protocols in the administration of antibiotics and in the treatment of severe type of periodontitis. More accurate approach might be as follows: to analyze the sub gingival micro flora, to characterize periodontopathogens, to decide for prescribing anti microbial regime or not, and to test that anti microbial drugs for bacterial resistance and susceptibility. Otherwise, antibiotics are not always clinically effective. Nevertheless, in most of the studies, bacterial resistance and susceptibility are disregarded and bacterial counts are reported; following administration of any anti microbial regimes. The most optimal antibiotic regime would be determined with antibiotic susceptibility testing. On the other hand, different microbiology laboratory set-ups, hence different test reports, complex microbial flora, empiric and the misuse of antibiotics may limit to use of microbial test.

Having taken together all the provided data from other studies, it is difficult to compare and conclude adjunctive benefits of anti microbial treatments due to both the paucity of randomized controlled studies and the different methodologies of the studies. Moreover, suppression/eradication of targeted microorganisms does not mean to achieve clinical efficacy (9).

## Conclusions

Within the limitation of the present study, we conclude that: although both antibiotic regimes together with non surgical treatment provided similar and favorable end results at 6 months, moxifloxacin use could be preferred by both dentists and the patients due to compliance because of the reduced number of tablets and the single-dose per day, as well as in patients with allergies, intolerance, or lack of response to amoxicillin and metronidazole. Nevertheless, anti microbial treatment should be based on individual characteristics of the patients.

Further researches may involve individual risk assessments, and individual treatment protocols.
